# Comparison and Evaluation of Real-Time Taqman PCR for Detection and Quantification of Ebolavirus

**DOI:** 10.3390/v13081575

**Published:** 2021-08-10

**Authors:** Yi Huang, Shuqi Xiao, Zhiming Yuan

**Affiliations:** 1 National Biosafety Laboratory, Chinese Academy of Sciences, Wuhan 430020, China; 2 Wuhan Institute of Virology, Chinese Academy of Sciences, Wuhan 430020, China; xiaosq2017@163.com

**Keywords:** ebolavirus, detection, quantification, real-time TaqMan PCR, comparison, evaluation

## Abstract

Given that ebolavirus causes severe and frequently lethal disease, its rapid and accurate detection using available and validated methods is essential for controlling infection. Real-time reverse-transcription PCR (RT-PCR) has proven to be an invaluable tool for ebolaviruses diagnostics. Many assays with different targets have been developed, but they have not been externally compared or validated, and limits of detection are not uniformly reported. Here we compared and evaluated the sensitivity, reproducibility and specificity of 23 in-house assays under the same conditions. Our results showed that these assays were highly gene- and species- specific when evaluated using in vitro RNA transcripts and viral RNA, and the potential limits of detection were uniformly reported ranging from 10^2^ to 10^6^ in vitro synthesized RNA transcripts copies perμL and 1–100 TCID_50_/mL. The comparison of these assays indicated that those targeting the more conservative NP gene could be the better option for EVD case definition and quantitative measurement because of its higher sensitivity for the same species. Our analysis could contribute to the standardization of ebolavirus detection and quantification assays, which can offer a better understanding of the meaning of results across laboratories and time points, as well as make them easy to implement, especially under outbreak conditions.

## 1. Introduction

The Ebolavirus genus falls within the *Filoviridae* family and *Mononegavirales* order, and it constitutes enveloped, non-segmented and negative-polarity RNA viruses [1]. Six distinct species have been identified in the *Ebolavirus genus* (ebolaviruses): *Zaire ebolavirus* (Ebola virus, EBOV), *Sudan ebolavirus* (Sudan virus, SUDV), *Reston ebolavirus* (Reston virus, RESTV), *Taï Forest ebolavirus* (Taï Forest virus, TAFV), *Bundibugyo ebolavirus* (Bundibugyo virus, BDBV) and *Bombali* *ebolavirus* (Bombali virus, BOMV) [2,3]. EBOV has been responsible for the most epidemics, for example, Makona, one of its recognized variants, was the causative agent in the 2014–2016 West Africa epidemic which affected approximately 30,000 individuals and claimed over 11,000 lives [4]. Prior to this epidemic, 13 outbreaks with a total of 1460 cases of EBOV infections were documented following its discovery in 1976 [5]. The most recent outbreak in the Democratic Republic of Congo, which is ongoing at the time of writing [6], has claimed more than 3000 lives and is the largest EBOV outbreak in the nation’s history. A new EBOV variant has been implicated as the cause of the outbreak with the proposed name “Tumba” [7].

Ebola virus disease (EVD) is a lethal viral hemorrhagic fever caused by ebolavirus and has a case fatality rate ranging from 25% to 90%. Clinically, EVD patients have non-specific flu-like symptoms along with hemorrhagic complications and multiple organ failure, which usually appear in the late stage of the disease. EVD symptoms usually include sudden onset of fever, muscle pain, headache and sore throat, followed by vomiting, diarrhea, rash and both internal and external bleeding (e.g., blood in stools and bleeding from the gums) [1]. The incubation period of EVD in humans varies, symptoms characteristically begin from 3 to 13 days after exposure [8]. Even though two vaccines: Ervebo (rVSV-ZEBOV) and a two-dose combination of Zabdeno (Ad26. ZEBOV) and Mvabea (MVA-BN-Filo) have been licensed in response to the devastating outbreak in recent years [9]. The licensing of an ebolavirus vaccine for use is challenging for several reasons. Successful control of the outbreak depended on prevention of transmission through rapid disease detection, based on symptoms, timely patient isolation and other important strategics components as outlined by WHO [9,10].

The diagnostics of EVD based solely on clinical grounds is very difficult. This is due to the infrequency of hemorrhagic symptoms, the non-specificity of above-described symptoms which usually mark the early onset of the disease, and the list of possible differential diagnoses such as malaria, typhoid fever, leptospirosis, diarrhea and other viral hemorrhagic fevers associated with flaviviruses (yellow fever and dengue), arenaviruses (Lassa fever), bunyaviruses (Rift Valley fever, Crimean-Congo hemorrhagic fever) and other filoviruses (Marburg virus disease) [11,12]. Moreover, the lengthy silent period of EVD can create difficulties in halting transmission and identifying the disease. The last 5 years have seen an unprecedented number of cases of EVD, which has taken an enormous toll in terms of mortality, economic damage, disruption to other public health programs and infrastructure, and public fear and mistrust [13]. The ease of international travel has increased the potential for transmission on an epidemic scale. Early detection of outbreaks is critical to a timely response.

Several diagnostic methods are available for the detection and identification of -ebolaviruses including virus isolation, enzyme-linked immunosorbent assays for detection of antigens or antibodies, reverse transcription polymerase chain reaction (RT-PCR) and electron microscopy, all of which have played major roles in the diagnosis of ebolaviruses infections. However, serological tests are not particularly useful in diagnosing acute ebolaviruses infection, because the presence of the corresponding IgG widespread in an ebolaviruses-endemic area (in recovered patients) and the corresponding IgM can present in different stages of EVD. Therefore, diagnosing recent ebolaviruses infection might require sequential blood draws to ascertain increasing IgM titres [14]. Several rapid antigen detection tests have also been developed, such as ReEBOV, SD Q Line and OraQuick [15,16,17]. However, these tests have low sensitivity and specificity. Their results still require confirmation by PCR, and are, at best, semiquantitative. On the other hand, PCR testing has the advantages of rapid turnaround, high degree of accuracy and detection of low levels of nucleic acids originating from either live or non-viable pathogens. Most importantly, PCR tests can potentially detect the pathogen during the early stage of disease, even before an immune response is detectable and any clinical signs are present [18]. In recent years, real-time RT-PCR for the diagnostics of emerging virus infections has been considered a very useful tool for case identification and outbreak control, e.g., during the COVID-19 pandemic.

Many groups have developed real-time RT-PCR assays with different targets that have both high sensitivity and high-throughput capacity [19,20,21,22,23,24]. Real-time RT-PCR is currently the benchmark method for EVD diagnostics [25]. However, most of these assays have not been externally standardized or validated, and limits of detection are not uniformly reported; for instance, they can be expressed as plaque-forming units, 50% tissue culture infectious dose per milliliter (TCID_50_/mL) or copies per mL [20,24,26,27]. The data gathered from different laboratories thus cannot be compared, because each laboratory uses distinct and specific assays under variable conditions. In addition, regulatory hurdles involved in validating assays and the urgent need for rapid EVD diagnostics have prompted the development of validated quantitative assays during the outbreak. Consequently, a comparison of currently available real-time RT-PCR assays is of paramount importance, particularly for emergency situations.

Therefore, we compared the sensitivity, reproducibility and specificity of 23 in house assays for the detection and quantification of mimic and viral RNA samples of Ebola virus to offer accessible references for standardization and assessment of these assays. The analytical specificity and sensitivity of each assay was evaluated using in vitro synthesized viral RNA transcripts. EBOV specific assays were also evaluated using viral RNAs extracted from cell-culture-propagated viruses, confirming that they are gene- and species specific. Thus far, only a few papers have been published that are directly related to comprehensive validation and comparison of these assays as a diagnostic tool.

## 2. Materials and Methods

### 2.1. Primers and Probes

A literature review was carried out to collect 23 real time RT-PCR assays for ebolaviruses detection (Table 1). The regions of NP, GP and VP40 are targeted for primers and probes. 6-carboxy-fluorescein (FAM) was the fluorescent reporter dye that covalently linked to its 5′ end. Furthermore, the quencher dye was Black Hole Quencher^®^-1(BHQ1) covalently attached to its 3′ end. All primers and probes were synthesized by AUGCT Biotechnology (Wuhan, China).

### 2.2. Cells and Viruses

Ebolaviruses Mayinga and Makona variant are provided by National Biosafety Laboratory, Chinese Academy of Sciences. The Vero E6 cell line and 293T cell line was obtained from the Preservation Center in Wuhan Institute of Virology, Chinese Academy of Sciences. Virus stocks were propagated in Vero E6 cells, and infectious titers determined by 50% tissue culture infectious dose assay. All work with infectious Ebola viruses were performed in BSL-4 facility of National Biosafety Laboratory in Wuhan, China, which had been certified by the China National Accreditation Service for Conformity Assessment (CNAS) and the National Health and Family Planning Commission of China [37]. Cells (293T) were transfected with recombinant pCMV-C-His containing ebolaviruses NP, GP and VP40 gene using Lipofectamine 2000 (Invitrogen, Carlsbad, CA, USA) according to the manufacturer’s instructions.

### 2.3. Virus Titration

Viruses were titrated by TCID_50_ on Vero E6 cells. Briefly, 96-well plates containing Vero E6 cells were incubated for 1 h at 37 °C in a 5% CO_2_ incubator with 0.1 mL of serial dilutions of virus stocks using 1:10 as the starting dilution. Add 100 uL of 2% FBS medium in each well. After incubation for 5–7 days at 37 °C in 5% CO_2_, CPE was observed and calculate the TCID_50_/mL.

### 2.4. RNA Isolation

Viral RNA was extracted from 140 μL of supernatant from virus-infected Vero cells using the RNA extraction kit (QIAamp Viral RNA Mini Kit, Qiagen Inc., Valencia, CA, USA) following the manufacturer’s instructions. The extracts were resuspended in 60 μL of Buffer AVE, aliquoted and stored at −70 °C before RT-PCR amplification was carried out. Total RNAs from transfected 293T cells were isolated using E.Z.N.A.^TM^ Total RNA Kit I (Omega Bio-tek, Norcross, GA, USA) according to the manufacturer’s instructions as mimic viral RNA for specificity evaluation of assays.

### 2.5. Preparation of In Vitro RNA Transcripts

The ebolaviruses in vitro RNA transcripts were synthesized using the T7 RiboMAX^TM^ expression large-scale RNA production system (Promega, Madison, WI, USA) according to the manufacturer’s instructions. Briefly, the high-quality DNA templates (plasmid containing the target gene including NP, GP or VP40 of different Ebola virus species) were linearized by restriction enzyme (TaKaRa Bio, Shiga, Japan) and then purified by E.Z.N.A.TM Cycle-Pure Kit (Omega). The in vitro transcription reactions were carried out 30 min at 37 °C with 2 μg DNA. Two units of RQ1 RNase-free DNase (Promega) were then added and incubation was continued for a further 60 min. The reactions were incubated for 15 min at 70 °C to inactivate the DNase. The transcripts were extracted using an E.Z.N.A.^TM^ MicroElute RNA Clean-up Kit (Omega) and resuspended in 50 μL of DEPC-treated water. The concentration of RNA transcript was determined by NanoDrop 2000 Spectrophotometer (Thermo Scientific, Wilmington, DE, USA). RNA transcripts were stored at −70 °C throughout this study.

### 2.6. Real-Time One-Step RT-PCR Analysis

The primers and probes used in these assays were listed in Table 1. One-step Real-time quantified RT-PCR assays were optimized using AgPath-IDTM One-step RT-PCR Kit (Applied Biosystems, Carlsbad, CA, USA). According to the user guide, each reaction contained 12.5 μL 2xRT-PCR Buffer, 1 μL Forward primer (10 μM), 1 μL Reverse primer (10 μM), 0.3 μL TaqMan probe (10 μM), 1 μL 25xRT-PCR Enzyme Mix, 0.05 μL RNase Inhibitor (40 U/μL, Beyotime, Shanghai, China), 7.15 μL Nuclease-free Water and 2 μL RNA. In place of sample, the extraction from non-infected cell culture supernatant and Nuclease-free Water was utilized as negative control. One-step Real-time qRT-PCR amplification was performed on a Bio-Rad CFX96 system. The procedure was as follow: reverse transcription at 4 °C for 10 min, RT initial denaturation at 95 °C for 10 min, 40 cycles of amplification at 95 °C for 15 s and annealing/extension at 60 °C for 45 s.

## 3. Results

### 3.1. Synthetic RNA Transcript Standards for Real-Time One-Step RT-PCR Assays

The sensitivity of real-time RT-PCR ebolaviruses specific assays was evaluated firstly using in vitro RNA transcripts, each containing one of the ebolaviruses NP, GP and VP40 coding sequences (CDS). The quality of the RNA standards synthesized by the in vitro T7 RNA transcription system was good enough for real-time RT-PCR assays because the ratios of absorbance at 260 and 280 nm (A_260_/A_280_) were between 2.0 and 2.1 (indicating high purity), and the concentrations of the RNA transcripts ranged from 1 to 7 mg/μL. RNA copy numbers were calculated according to the concentration and molecular weight of each single-stranded RNA fragment. The threshold cycle (Ct) values were calculated from the various amplification plots of the range of dilutions and used to draw a standard curve which could easily be used for an absolute quantitative analysis of unknown samples (Table 2 and Table S1, Supplementary Materials).

### 3.2. Detection Limits of the Assays

In this case, 23 sets of primers and probes targeting the NP, GP and VP40 genes of the ebolaviruses RNA genome were used for the specific amplification of each of the 5 ebolavirus species (Table 1). The sensitivity of real-time RT-PCR assays for different ebolavirus species was firstly determined using serial dilutions of known amounts of in vitro RNA transcripts containing the CDS of NP, GP or VP40 from each species of ebolaviruses. Ct values of the range of dilutions covering 7–9 serially diluted RNA transcripts were used to draw standard curves. Each corresponding copy number was calculated based on the molecular weight of the target sequence. The potential detection limits for each species-specific assay were determined to be 1 to 10^4^ RNA copies/μL for EBOV, 10^4^–10^5^ RNA copies/μL for SUDV, 10^3^–10^6^ RNA copies/μL for TAFV, 10^5^ RNA copies/μL for BDBV and 10^4^ RNA copies/μL for RESTV (Table 2). Among them, the potential detection limits for NP gene specific assays were 1–10^5^ RNA copies/μL. While those for other gene-specific assays (GP and VP40) were higher (10^2^–10^6^ RNA copies/μL). In particular, the potential detection limits for EBOV NP gene specific assays were 1–10 RNA copies/μL, while those for SUDV, TAFV and BDBV NP gene specific assays were much higher (10^5^ RNA copies/μL. Similarly, the potential detection limits for EBOV GP-gene specific assays (10^2^–10^3^ RNA copies/μL) were also lower than GP-gene specific assays for other species (10^4^–10^6^ RNA copies/μL). After a summarization, we found that there were more assays for EBOV than for other species. In conclusion, the most sensitive among the 23 sets were those specific for EBOV NP(ZENP-W and ZSENP-Z), and the assays specific for the NP gene were generally the most sensitive also in the case of other ebolavirus species. No inhibition of the assays at high concentrations of RNA (10^12^ copies/μL).

Next, the sensitivity of the real-time RT-PCR assays was evaluated using the viral RNA extracted from the serial dilutions of known titre of virus stock (Table 3). The potential detection limits for each assay were 1–100 TCID_50_/mL (where TCID_50_ is the 50% tissue culture infective dose). All tests were repeated more than three times and the inter-assay coefficients of variation (CVs) of Ct values were within the acceptable limit (<3%), indicating their reproducibility.

The potential detection limits of ebolaviruses specific assays expressed in vitro RNA transcripts per microliter and 50% tissue culture infectious dose per milliliter (TCID_50_/mL) are summarized and compared in Table 4. The sensitivities of the EBOV specific assays were consistent when expressed in both different units of measure. For example, ZENP-W, specific for EBOV NP, was the most sensitive assay and the potential detection limits for ZEGP-R were the highest among those specific for EBOV GP, whether evaluated using in vitro RNA transcripts (10^0^ RNA copies/μL for ZENP-W and 10^6^ RNA copies/μL for ZEGP-R) or viral RNA (1 TCID_50_/mL for ZENP-W and 100 TCID_50_/mL for ZEGP-R). The comparison also indicated different gene copy number for gene encoding different structural proteins in each virus particle. That is, if one copy of the NP gene was included in each infectious EBOV virus particle, there would be 10 times more GP gene copies and 1000 times more VP40 gene copies.

### 3.3. Specificity of the Assays

**Figure 1:**
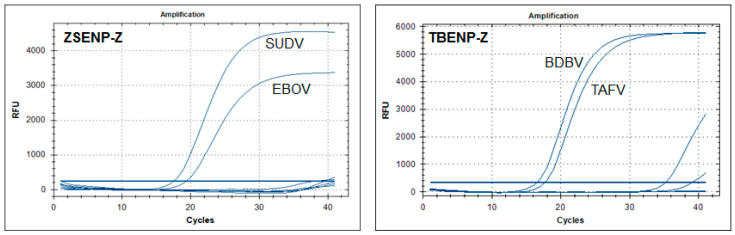
Specificities of the ZBENP-Z and TBENP-Z assays were examined using a variety of mimic viral RNA including Marburg virus (MARV), SUDV, TAFV, BDBV and RESTV. They were designed to be simultaneously specific for two EBOV species: ZSENP-Z for EBOV and SUDV, whereas TBENP-Z for TAFV and BDBV RFU: relative fluorescence units.

To evaluate the specificity of these assays, cross-reactivity was examined using a variety of mimic viral RNA including Marburg virus (MARV), SUDV, TAFV, BDBV and RESTV (Table S1, Supplementary Materials). Marburg viruses can either cause similar symptomatology to ebolaviruses or could be a possible differential diagnosis to EVD. Each set showed highly positive fluorescence signals in the Ct range of 11.99–22.21 according to its own template, i.e., the RNA extracted from cells transfected with a specific gene expression plasmid. No cross-amplification reactions were observed. ZSENP-Z and TBENP-Z were designed to be simultaneously specific for two EBOV species. ZSENP-Z for EBOV and SUDV, whereas TBENP-Z for TAFV and BDBV (Table 5 and Figure 1).

**Figure 2:**
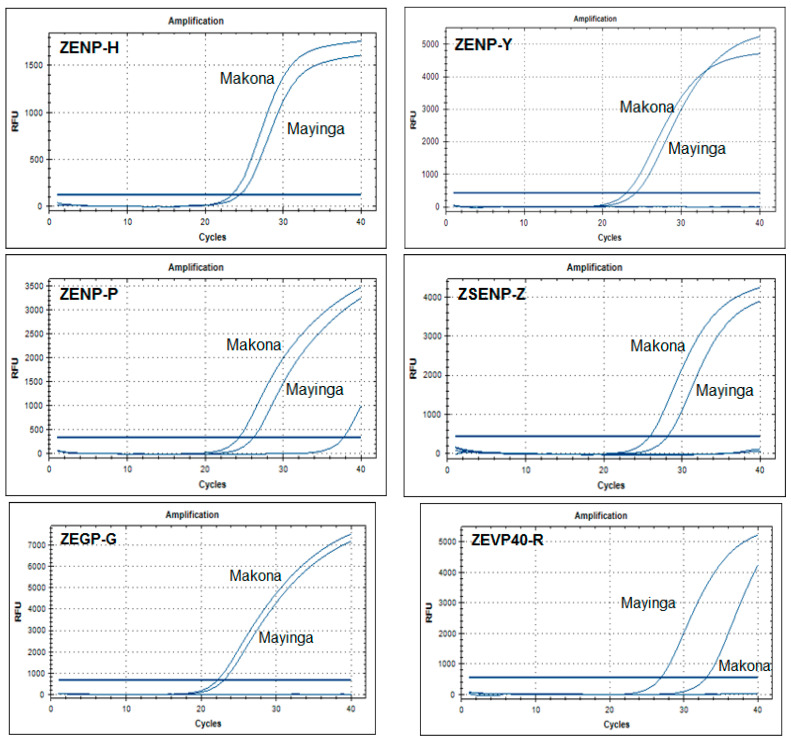
Specificity of ZENP-H, ZENP-Y, ZSENP-Z and ZEVO40-R assays for the EBOV Mayinga variant and the Makona variant. The specificity of ebolavirus species-specific real-time RT-PCR assays was examined using viral RNA extracted from the supernatant of cells infected with the EBOV-Mayinga variant and the Makona variant. Outside these assays, there was no difference in assay specificity between the two variants.

Most EBOV species-specific primer sets were designed using the consensus sequence derived from available sequences in GenBank. This was before the EVD outbreak in West Africa, and some mismatches were found between several EBOV variants and Makona variants, which were responsible for this West Africa outbreak. Fortunately, our tests indicated that most of EBOV-specific assays were also specific for the Makona variant (Table 6 and Table S2, Supplementary Materials). Even though there were some differences in specificity for these two variants among four EBOV NP-specific assays (ZENP-H, ZENP-Y, ZENP-P and ZSENP-Z (*p* < 0.05), and significant difference between the other two assays: ZEGP-G and ZEVP40-R (*p* < 0.01) see Table 6 and Figure 2). What need to be concerned was that the potential detection limit of the ZEVP40-R assay for the Makona variant was significantly higher than those for the Mayinga variant (Table 6 and Figure 2). This could be due to the several amino-acid changes in the Makona variant VP40 protein, which are conserved among most other EBOV isolates. Thus, it is recommended to develop a more specific assay targeted at the VP40 gene for the Makona variant.

## 4. Discussion

The two deadliest ebolaviruses epidemics have occurred in the past 5 years, and one of these epidemics is still ongoing. In the case of an epidemic emergency, the first line of response should be an accurate and quick virus detection. Nucleic acid detection is the most common procedure for diagnosing EVD because of its unsurpassed specificity and sensitivity, as well as its ability to detect acute infection. Additionally, the virus does not need to be viable at the time of testing [38]. In recent years, real-time RT-PCR for the diagnostics of emerging virus infection has proven to be an invaluable tool for case identification and epidemic control [39,40,41], e.g., during the ongoing COVID-19 pandemic. In addition, when evaluating the spread of an infectious disease, the deployment of any diagnostic effort must rely on available and validated methods because time is very limited for research or the development of new technologies in the context of an emergency. Here, we comprehensively summarized and compared the sensitivity, reproducibility, and specificity of 23 in house assays, thus contributing to the standardization of ebolavirus detection and quantification assays.

Our summarization showed that more assays for EBOV (11 sets) than other ebolaviruses species as it has been responsible for the most epidemics and the highest fatality rate (Table 1). We also showed the higher sensitivity for EBOV, especially for the EBOV NP gene target (Table 2). For example, ZSENP-Z, which was specific for EBOV and SUDV, had sufficient specificity, but its potential detection limit for SUDV (10^5^ RNA copies/μL) was much higher than that for EBOV (10^0^ RNA copies/μL), which would likely SUDV being overlooked when simultaneously detecting two viruses. Therefore, additional assays for other ebolaviruses species (SUDV, TAFV, BDBV and RESTV) with higher sensitivity and specificity should be developed in the future. In addition, assays specific for different EBOV variants should also be developed for future possible outbreaks, such as the Makona variant, which is responsible for the EBOV outbreak in West Africa and the ongoing outbreak in the Democratic Republic of Congo. In this study, we showed that some EBOV-specific assays (such as ZEVP40-R) were not sufficient to detect the Makona variant. Even though the conserved NP gene was shown a good target for sensitive and specific assays, we should also consider the different specificities of assays for different regions of this gene. For example, the specificities of ZENP-Wand ZENP-L were better than those of ZENP-H, ZENP-Y, ZSENP-Z and ZENP-P, whereby the latter targeted the region near the 5′ end of the NP gene (in reference to the EBOV sequence NC_002549).

The quantitative measurement of viral load is an important parameter in the management of EVD because viral load correlates with severity of disease, survival and infectivity [26,42,43,44,45,46,47]. Viral load measurements are also important to interpret the efficacy of candidate therapies and vaccines in animal models and human beings [42,44], as well as to interpret ebolaviruses persistence in and transmission risk from immune privileged body compartments and fluids, such as the male gonads or semen, eyes, CNS, breast milk and the intrauterine space in pregnant women [48,49,50,51,52]. Additionally, ebolaviruses have occasionally been found in sweat and urine [53], and in atypical or asymptomatic cases [52]. Viral load measurement could even be useful in assessing environmental decontamination practices [54]. As known, real-time RT-PCR not only provides a qualitative diagnosis but is also a direct measure of the virus load in a sample via determining the cycle threshold. For example, during the ongoing EVD outbreak in parts of Western Africa, more than 44 diagnostic laboratories yielded qualitative results for the detection of EVD [55].

Even though several real-time RT-PCR assays for Ebola virus are commercially available, none of them have been validated for quantitative viral load assessment. The reported sensitivities of these assays vary substantially depending on the reagents and other materials. Moreover, various in-house quantitative assays for detecting the viral load of ebolaviruses have been described, as summarized in this study, however, most of these assays have not been externally standardized or validated. Limits of detection depend on the PCR platform used and are not uniformly reported. Thus, data gathered between laboratories generally cannot be compared, because each laboratory possibly used distinct and specific assays under variable conditions [56]. A 1–2 log10 difference in viral load may be within the margin of error of RT-PCR testing across and within laboratories and assays. Here 23 assays for ebolavirus were compared using good research laboratory practices for the quantitative measurement of viral load in virus stock using the same synthetic RNA standard (RNA copies/μL), which could be used to standardize results across laboratories and time points. Furthermore, 11 sets for EBOV among them were further evaluated and compared using serial dilutions of known titre of virus stock (TCID_50_/mL). Our analysis showed that there was no substantial difference in the quantitative measurement of viable viral load across the two measures of potential detection limits. However, it will be easier and safer for these assays to implement synthetic RNA as a quantitative measurement standard (RNA copies/μL) in resource-limited settings and under outbreak conditions. As the viral load does not necessarily correlate with viable replicating ebolavirus, RNA copies per μL was more applicable for use in environmental decontamination assessment than TCID_50_/mL.

Quantitative assays are labor intensive and not very easy to implement under field conditions, while the results might not be universally replicable, because they could vary depending on different reagents, machines and technician experience. Viral load determination has become a routine test for some virus diseases, such as HIV/AIDS and SARS-CoV-2/COVID-19, and it has the basis of clinical patient management. A path forward might be gleaned from concerted international efforts to develop standardized quantitative assays and reference materials. Virologists could work at the international level to standardize techniques and validate a robust field reference [37,57]. This is another important concern that we addressed here by standardizing 23 assays for different ebolaviruses species under the same conditions, i.e., the same reagent, the same machine. We uniformly reported the potential detection limits as RNA copies per μL and TCID_50_ per mL. We believe that these results could contribute to the standardization and validation of assays for ebolaviruses detection and quantification, as well as enable rapid, accurate and easy-to-implement EVD diagnostic methods in resource-limited settings and under outbreak conditions. Of course, the commutability of some materials might need additional work to yield consensus, and these efforts are clearly expected to have a positive effect on assay comparability. All efforts toward standardizing ebolaviruses assays hold the promise of similar effects and should be vigorously pursued.

## Figures and Tables

**Table 1:** Real-time One-step RT-PCR assay summary.

Virus Species	Target	Assay	Amplicon Size (bp)	Reference
EBOV	NP	ZENP-H	80	[22]
		ZENP-W	70	[28]
		ZENP-Y	161	[29]
		ZENP-P	133	[30]
		ZENP-L	161	[31]
	GP	ZEGP-T	80	[24]
		ZEGP-R	141	[32]
		ZEGP-G	112	[21]
		ZEGP-L	145	[33]
	VP40	ZEVP40-R	161	[32]
SUDV	NP	SENP-T04	69	[26]
		SENP-T08	74	[34]
		SENP-P	103	[30]
		SENP-W	70	[28]
	GP	SEGP-T	77	[24]
		SEGP-G	111	[21]
EBOV and SUDV	NP	ZSENP-Z	123	[35]
TAFV	NP	TENP-P	88	[30]
	GP	TEGP-T	79	[24]
	VP40	TEVP40-D	97	[36]
BDBV	NP	BENP-T	75	[34]
TAFV and BDBV	NP	TBENP-Z	77	[35]
RESTV	VP40	REVP40-T	80	[24]

**Table 2:** The standard curves and detection limits of TaqMan RT-PCR assays for different ebolavirus species. Amplification plots were realized on 10-fold dilutions of known concentration of transcript RNAs containing the CDS of NP, GP or VP40 from each species of ebolavirus. From the obtained standard curves, the sensitivity for each assay was also determined. (*x* stand for Log copies/μL and *y* stand for Ct value).

Assay	Standard Curve	Limits of Detection
ZENP-H	y = −3.3488x + 40.203 R^2^ = 1	10^1^ RNA copies/μL
ZENP-W	y = −3.1843x + 38.867 R^2^ = 1	10^0^ RNA copies/μL
ZENP-Y	y = −3.4368x + 41.094 R^2^ = 1	10^1^ RNA copies/μL
ZENP-P	y = −3.6168x + 42.187 R^2^ = 1	10^1^ RNA copies/μL
ZENP-L	Y = −3.4958x + 41.038 R^2^ = 1	10^1^ RNA copies/μL
ZSENP-Z	y = −3.2535x + 40.697 R^2^ = 1	10^1^ RNA copies/μL (EBOV)
ZEGP-T	y = −3.6921x + 46.095 R^2^ = 1	10^2^ RNA copies/μL
ZEGP-R	y = −3.3082x + 47.782 R^2^ = 1	10^3^ RNA copies/μL
ZEGP-G	y = −3.2236x + 45.698 R^2^ = 1	10^2^ RNA copies/μL
ZEGP-L	y = −3.3500x + 46.64 R^2^ = 1	10^2^ RNA copies/μL
ZEVP40-R	y = −3.7963x + 52.524 R^2^ = 1	10^4^ RNA copies/μL
SENP-T04	y = −3.5607x + 55.573 R^2^ = 1	10^5^ RNA copies/μL
SENP-T08	y = −3.4582x + 54.547 R^2^ = 1	10^5^ RNA copies/μL
SENP-P	y = −3.4264x + 54.151 R^2^ = 1	10^5^ RNA copies/μL
SENP-W	y = −3.4850x + 54.82 R^2^ = 1	10^5^ RNA copies/μL
ZSENP-Z	y = −3.4364x + 55.501 R^2^ = 1	10^5^ RNA copies/μL (SUDV)
SEGP-T	y = −3.1054x + 52.319 R^2^ = 1	10^4^ RNA copies/μL
SEGP-G	y = −3.1118x + 52.05 R^2^ = 1	10^4^ RNA copies/μL
TENP-P	y = −3.1736x + 53.329 R^2^ = 1	10^5^ RNA copies/μL
TBENP-Z	y = −3.3521x + 56.003 R^2^ = 1	10^5^ RNA copies/μL (TAFV)
TEGP-T	y = −3.1179x + 56.335 R^2^ = 1	10^6^ RNA copies/μL
TEVP40-D	y = −3.3279x + 47.471 R^2^ = 1	10^3^ RNA copies/μL
BENP-T	y = −3.5357x + 55.8 R^2^ = 1	10^5^ RNA copies/μL
TBENP-Z	y = −3.6682x + 57.733 R^2^ = 1	10^5^ RNA copies/μL (BDBV)
REVP40-T	y = −3.0955x + 52.095 R^2^ = 1	10^4^ RNA copies/μL

**Table 3:** The detection limits of the TaqMan RT-PCR assays for EBOV. The sensitivity of EBOV real-time RT-PCR assays was evaluated using viral RNA extracted from serial dilutions of known titre of virus stock. Amplification plots were realized on viral RNA extracted from 10-fold dilutions (10^−1^~10^−7^ dilution, i.e., 10^6^~10^0^ TCID50/mL) of virus-infected cell supernatants.

Assay	Standard Curve	Limits of Detection
ZENP-H	y = −3136x + 40.161 R^2^ = 1	10 TCID_50_/mL
ZENP-W	y = −3.21x + 38.61 R^2^ = 1	1 TCID50/mL
ZENP-Y	y = −3.4654x + 41.854 R^2^ = 1	10 TCID_50_/mL
ZENP-P	y = −3.24x + 40.57 R^2^ = 1	10 TCID_50_/mL
ZENP-L	y = −3.3863x + 40.029 R^2^ = 1	10 TCID_50_/mL
ZSENP-Z	y = −3.49x + 41.96 R^2^ = 1	10 TCID_50_/mL
ZEGP-T	y = −3.4126x + 40.644 R^2^ = 1	10 TCID_50_/mL
ZEGP-R	y = −3.375x + 44.938 R^2^ = 1	100 TCID_50_/mL
ZEGP-G	y = −3.4177x + 40.559 R^2^ = 1	10 TCID_50_/mL
ZEGP-L	y = −3.3537x + 41.115 R^2^ = 1	10 TCID_50_/mL
ZEVP40-R	y = −3.4574x + 41.346 R^2^ = 1	10 TCID_50_/mL

**Table 4:** Summary and comparison of potential detection limits for EBOV specific real-time one-step RT-PCR assays reported as in vitro RNA transcripts per microliter and 50% tissue culture infectious dose per milliliter (TCID_50_/mL).

Assay	In Vitro RNA Transcripts	Viral RNA
ZENP-H	10 RNA copies/μL	10 TCID_50_/mL
ZENP-W	1 RNA copies/μL	1 TCID_50_/mL
ZENP-Y	10 RNA copies/μL	10 TCID_50_/mL
ZENP-P	10 RNA copies/μL	10 TCID_50_/mL
ZENP-L	10 RNA copies/μL	10 TCID_50_/mL
ZSENP-Z	10 RNA copies/μL	10 TCID_50_/mL
ZEGP-T	10^2^ RNA copies/μL	10 TCID_50_/mL
ZEGP-R	10^3^ RNA copies/μL	100 TCID_50_/mL
ZEGP-G	10^2^ RNA copies/μL	10 TCID_50_/mL
ZEGP-L	10^2^ RNA copies/μL	10 TCID_50_/mL
ZEVP40-R	10^4^ RNA copies/μL	10 TCID_50_/mL

**Table 5:** Specificity of ebolavirus species-specific real-time one-step RT-PCR assays.

Assay	Virus					
	EBOV	SUDV	TAFV	BDBV	RESTV	MARV
ZENP-H	+	−	−	−	−	−
ZENP-W	+	−	−	−	−	−
ZENP-Y	+	−	−	−	−	−
ZENP-P	+	−	−	−	−	−
ZENP-L	+	−	−	−	−	−
ZEGP-T	+	−	−	−	−	−
ZEGP-R	+	−	−	−	−	−
ZEGP-G	+	−	−	−	−	−
ZEGP-L	+	−	−	−	−	−
ZEVP40−R	+	−	−	−	−	−
ZSENP-Z	+	+	−	−	−	−
SENP-T04	−	+	−	−	−	−
SENP-T08	−	+	−	−	−	−
SENP-P	−	+	−	−	−	−
SENP-W	−	+	−	−	−	−
SEGP-T	−	+	−	−	−	−
SEGP-G	−	+	−	−	−	−
TENP-P	−	−	+	−	−	−
TBENP-Z	−	−	+	+	−	−
TEGP-T	−	−	+	−	−	−
TEVP40-D	−	−	+	−	−	−
BENP-T	−	−	−	+	−	−
REVP40-T	−	−	−	−	+	−

**Table 6:** Specificity of real-time one-step RT-PCR assays specific for the Makona variant. Viral RNA extracted from the supernatant of cells infected with the EBOV-Mayinga variants and the Makona variant was used and the Ct values of these two variants from each assay are shown. *, difference between the Mayinga variant and the Makona variant (*p* < 0.05); **, significant difference between the Mayinga variant and the Makona variant (*p* < 0.01).

Assay	Mean Ct Value	
	Mayinga	Makona
ZENP-H *	26.98	24.43
ZENP-W	23.36	23.25
ZENP-Y *	25.18	23.15
ZENP-P *	25.96	24.44
ZENP-L	24.62	23.45
ZEGP-T	21.22	21.45
ZEGP-R	23.02	23.01
ZEGP-G **	23.41	22.05
ZEGP-L	24.02	24.41
ZEVP40-R **	27.36	33.26
ZSENP-Z *	27.93	25.83

## Supplementary Materials

The following are available online at https://www.mdpi.com/article/10.3390/v13081575/s1, Table S1: The specificity of TaqMan RT-PCR assays for different Ebola virus species. The specificity was evaluated firstly by using the simulating viral RNA extracted from cells transfected with recombinant plasmid containing Ebola virus NP, GP and VP40 gene. Table S2: Specificity of EBOV species specific real-time RT-PCR assays for EBOV-Mayinga strain and Makona variant.
