# Supplementary material for: Comparison and Evaluation of Real-Time Taqman PCR for Detection and Quantification of Ebolavirus
Source: Viruses. 2021 Aug 10;13(8):1575. doi: 10.3390/v13081575 (PMC8402893; doi:10.3390/v13081575)

Supplementary

Table. S1 The specificity of TaqMan RT-PCR assays for different Ebola virus species. The specificity was evaluated firstly by using the simulating viral RNA extracted from cells transfected with recombinant plasmid containing Ebola virus NP, GP and VP40 gene.

Assay	Specificity	Assay	Specificity
ZENP-H	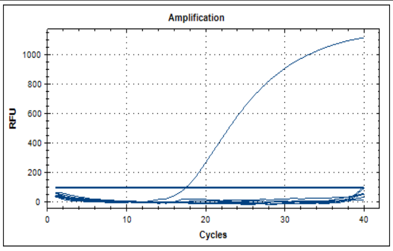	ZEGP-T	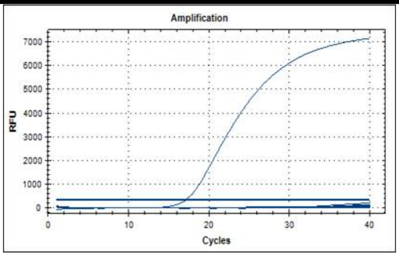
ZENP-W	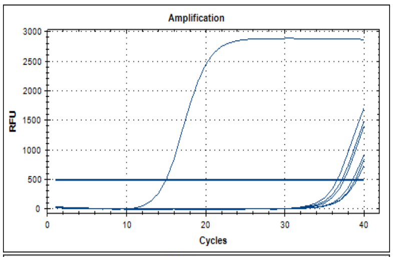	ZEGP-R	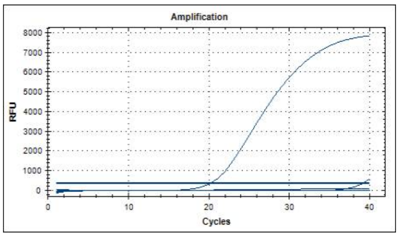
ZENP-Y	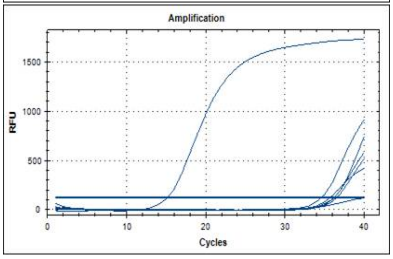	ZEGP-G	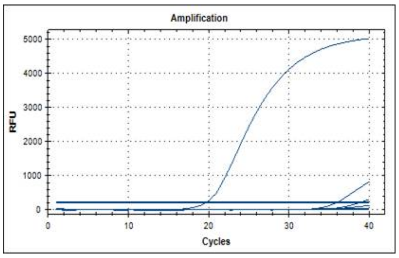
ZENP-P	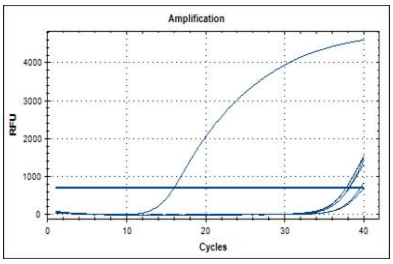	ZEGP-L	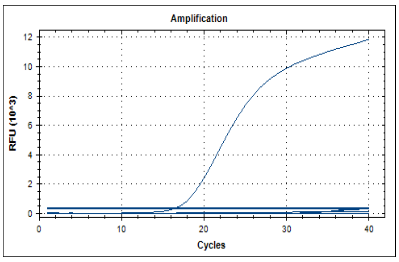
ZENP-L	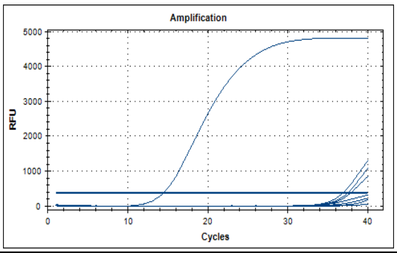	ZEVP40-R	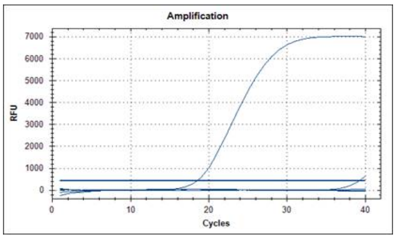

ZSENP-Z (EBOV)

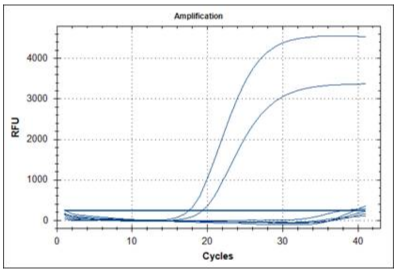

SEGP-T

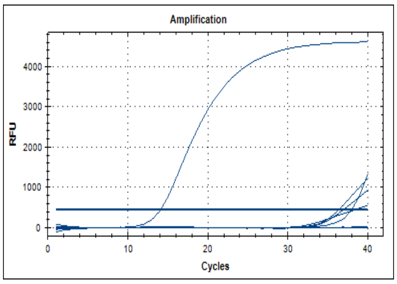

SENP-T04

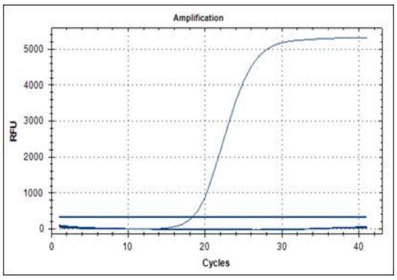

SEGP-G

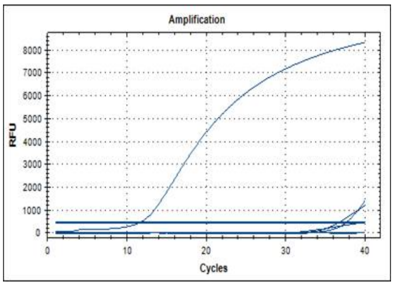

SENP-T08

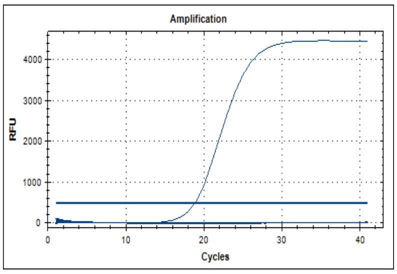

TEGP-T

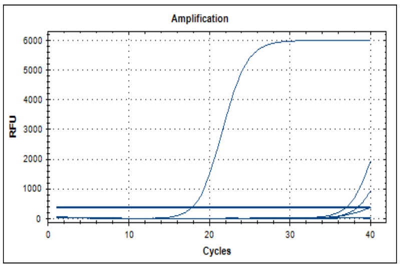

SENP-P

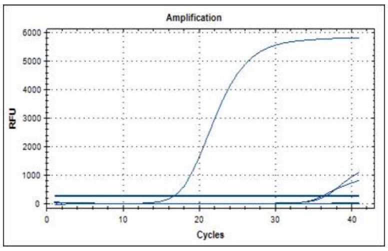

TEVP40-D

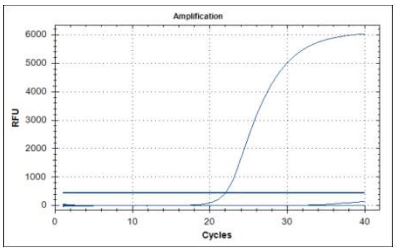

SENP-W

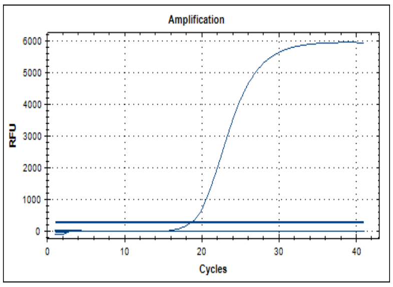

REVP40-T

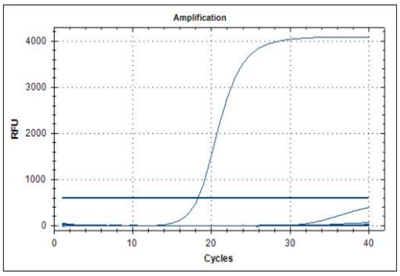

ZSENP-Z (SUDV)

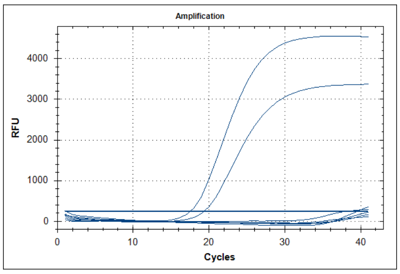

TENP-P

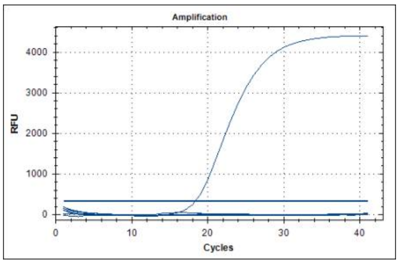

TBENP-Z (TAFV)	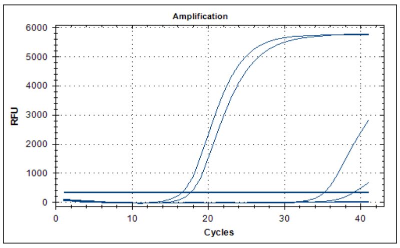	BENP-T	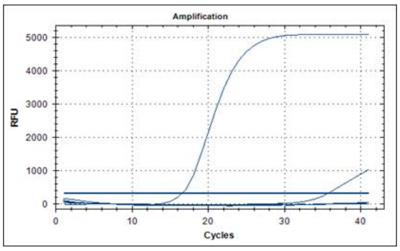
TBENP-Z (BDBV)	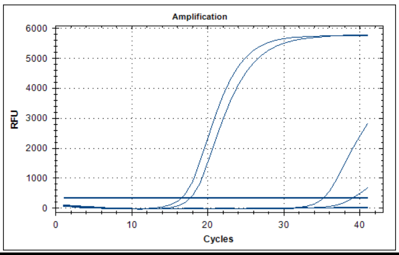		

Table S2. Specificity of EBOV species specific real-time RT-PCR assays for EBOV-Mayinga strain and Makona variant.

Assay	Specificity	Assay	Specificity
ZENP-H	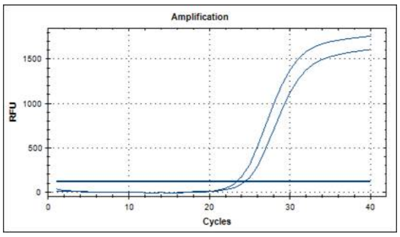	ZEGP-T	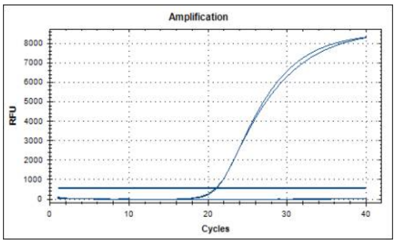
ZENP-W	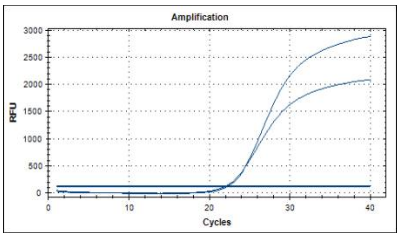	ZEGP-R	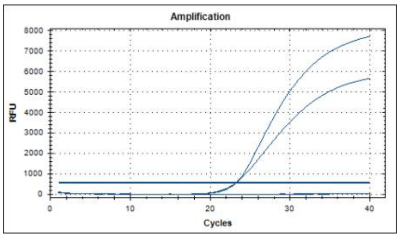
ZENP-Y	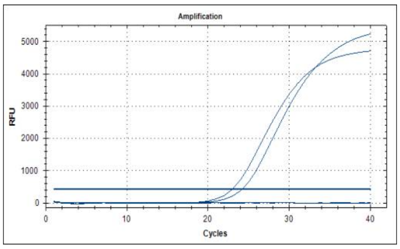	ZEGP-G	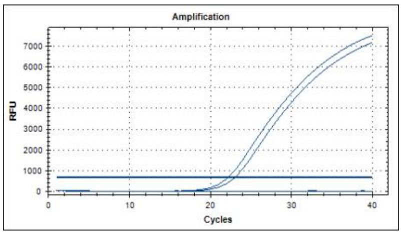
ZENP-P	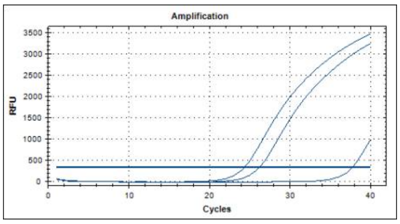	ZEGP-L	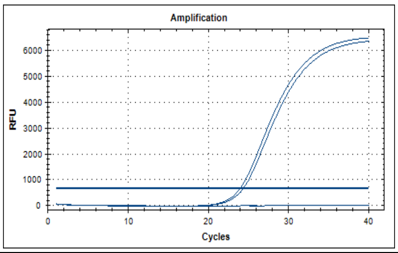

ZENP-L

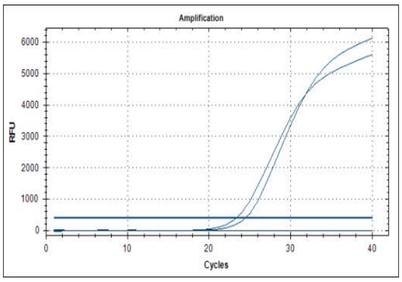

ZEVP40-R

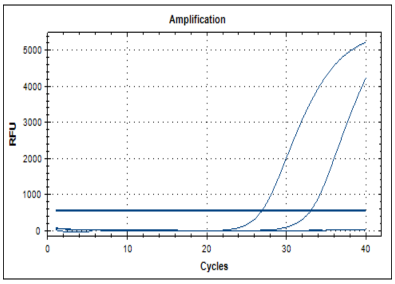

ZSENP-Z

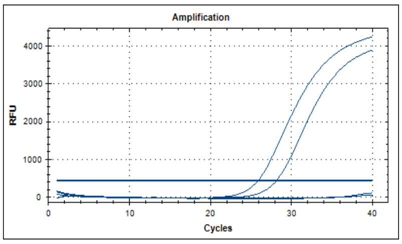

Supplement: Supplementary file 1 [file viruses-13-01575-s001.zip › viruses-1285994-supplementary.pdf]
